# Comparison of Ultrasonography Features and K-TIRADS for Isthmic and Lobar Papillary Thyroid Carcinomas: A Single-Center Study

**DOI:** 10.3389/fendo.2020.00328

**Published:** 2020-06-04

**Authors:** Yoo Jin Lee, Dong Wook Kim, Gi Won Shin, Jin Young Park, Hye Jung Choo, Ha Kyoung Park, Tae Kwun Ha, Do Hun Kim, Soo Jin Jung, Ji Sun Park, Sung Ho Moon, Ki Jung Ahn, Hye Jin Baek

**Affiliations:** ^1^Department of Radiology, Busan Paik Hospital, Inje University College of Medicine, Busan, South Korea; ^2^Department of General Surgery, Busan Paik Hospital, Inje University College of Medicine, Busan, South Korea; ^3^Department of Otorhinolaryngology-Head and Neck Surgery, Busan Paik Hospital, Inje University College of Medicine, Busan, South Korea; ^4^Department of Pathology, Busan Paik Hospital, Inje University College of Medicine, Busan, South Korea; ^5^Department of Nuclear Medicine, Busan Paik Hospital, Inje University College of Medicine, Busan, South Korea; ^6^Department of Anesthesiology and Pain Medicine, Busan Paik Hospital, Inje University College of Medicine, Busan, South Korea; ^7^Department of Radiation Oncology, Busan Paik Hospital, Inje University College of Medicine, Busan, South Korea; ^8^Department of Radiology, Gyeongsang National University Changwon Hospital, Gyeongsang National University School of Medicine, Changwon, South Korea

**Keywords:** thyroid, papillary thyroid carcinoma, isthmic, ultrasonography, K-TIRADS

## Abstract

**Objective:** This study aimed to compare ultrasonography (US) features and the Korean-Thyroid Imaging Reporting and Data System (K-TIRADS) categories for diagnosing isthmic and lobar papillary thyroid carcinomas (PTC).

**Methods:** From January 2009 to December 2012, 163 patients who underwent thyroid surgery and were confirmed with a post-operative histopathological diagnosis of isthmic PTC were retrospectively included. Fifty-nine patients were excluded because their tumor size was <0.5 cm or because of other reasons. The control group comprised of 145 patients who underwent thyroid surgery from January to April 2013 for a classic type of PTC, with the largest diameter being ≥ 0.5 cm and located in the thyroid lobe. A single radiologist retrospectively reviewed the US features and K-TIRADS categories of each nodule using a picture archiving and communication system.

**Results:** Among 104 patients with isthmic PTC, 95 and 9 had primary and secondary cancers, respectively. On the other hand, all 145 patients with lobar PTC had primary cancers. Isthmic PTC showed a lower prevalence of non-parallel orientation than lobar PTC (23.1 and 71%). Nodule orientation was the only US feature statistically different between the two groups (*p* < 0.0001). However, there was no significant difference in patient age, sex, nodule size, composition, echogenicity, microcalcification, spiculated/microlobulated margin, and K-TIRADS category between the two groups (*p* > 0.05).

**Conclusions:** K-TIRADS may be useful in the diagnosis of both isthmic and lobar PTC.

## Introduction

The thyroid isthmus is a small segment that connects both the thyroid lobes and located posterior to the strap muscles and anterior to the trachea (1, 2). A small proportion of papillary thyroid carcinomas (PTC) arise from the thyroid isthmus, and they are called as isthmic PTC (1, 2). Isthmic PTC reportedly tend to be more tend to be more aggressive than PTC of the thyroid lobe (called as lobar PTC), including more frequent lymph node metastasis, involvement of multiple foci, or local invasion next to the trachea and strap muscles (1–5). The extent of surgical treatment for isthmic PTC remains controversial, and no precise guidelines have been established for the management of patients with isthmic PTC (1–3, 5, 6).

Few studies comparing ultrasonography (US) features of isthmic and lobar PTC have been reported (7, 8). These studies described isthmic PTC as being a circumscribed mass with a wider-than-tall shape with more prominent extrathyroidal extension than lobar PTC (7, 8). Recently, the Korean Society of Thyroid Radiology proposed a new risk stratification system for diagnosis of thyroid nodules (i.e., the Korean Thyroid Imaging Reporting and Data System [K-TIRADS]) (9, 10). K-TIRADS considers three malignant US features (microcalcification, spiculated/microlobulated margin, and non-parallel orientation), and the solidity and echogenicity of the thyroid nodules. In a previous multicenter prospective validation study using K-TIRADS, the malignancy rates of K-TIRADS category 2, 3, 4, and 5 nodules were 0.0, 3.5, 19.0, and 73.4%, respectively (11). Therefore, K-TIRADS is a simple and effective tool for thyroid nodule evaluation. To our best knowledge, no studies comparing the K-TIRADS categories of isthmic and lobar PTC have been previously published. Therefore, the present study aimed to compare the US features and K-TIRADS categorization of isthmic and lobar PTC.

## Methods

### Study Population

This retrospective study was approved by the Institutional Review Board (IRB 19-0196), and the need for informed consent was waived. Among the 2,410 patients who underwent thyroid surgery for PTC at our institution from January 2009 to December 2012, 163 patients (141 female and 22 male; age range, 25–75 years; mean age ± standard deviation [SD], 46.9 ± 10.0 years) were confirmed with a post-operative histopathological diagnosis of a classic type of isthmic PTC. Among them, 59 patients were excluded because of the small tumor size (<0.5 cm, *n* = 23), lack of preoperative thyroid US images (*n* = 16), poor US image quality (*n* = 15), and uncertainty between US findings and histopathological results (*n* = 5). Eventually, 104 patients with isthmic PTC (88 female and 16 male; age range, 25–75 years; mean ± SD, 46.9 ± 9.9 years) were included in the study group. The control group comprised of 145 patients (127 female and 18 male; age range, 29–86 years; mean ± SD, 48.4 ± 10.9 years) who underwent thyroid surgery from January to April 2013 for a classic type of lobar PTC with the largest nodule diameter being ≥ 0.5 cm. The histopathological data were retrospectively investigated using patients' electronic medical records.

### Ultrasonographic Examination and Image Analysis

**Figure 1:**
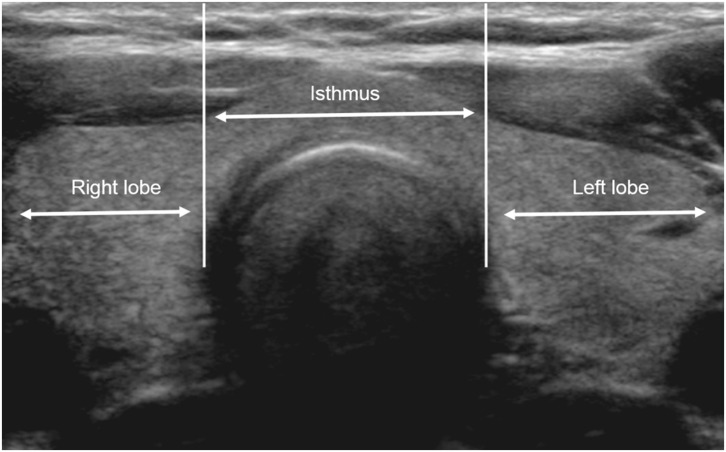
Boundary of the thyroid isthmus on ultrasonography (US). The lateral border of the isthmus is defined by two imaginary lines on transverse US images perpendicular to the skin surface from the most lateral tracheal wall. Thyroid nodules are classified as being of isthmic or lobar origin according to the location of its larger portion.

Preoperatively, thyroid or neck US was performed using a high-resolution ultrasound scanner (HDI 5000 or iU 22; Philips Medical Systems, Bothell, WA, USA) with a 5–12-MHz linear probe by an experienced radiologist. In this study, on transverse US images, the lateral border of the thyroid isthmus was defined by drawing two imaginary lines perpendicular to the skin surface from the most lateral points of the trachea (Figure 1). Thyroid mass located between these two imaginary lines was classified as being of isthmic origin. When the center of the PTC was located between these two imaginary lines, it was classified as an isthmic PTC.

The same radiologist retrospectively examined the US features and K-TIRADS category of each nodule using a picture archiving and communication system (9). The US features of the PTC included nodule composition (solid, cystic, or mixed), echogenicity (iso-, hypo-, and hyper-echogenic), calcification (microcalcification, macrocalcification, or rim calcification), margin (smooth, spiculated/microlobulated, or ill-defined), and orientation (parallel or non-parallel) (9). Nodule size was calculated from the US images. Based on the K-TIRADS classification, each nodule was classified as category 2 (benign), 3 (low suspicion), 4 (intermediate suspicion), or 5 (high suspicion), according to the solidity, echogenicity, and three malignant US features (microcalcification, spiculated/microlobulated margin, and non-parallel orientation) (9).

Regarding cases of multifocal PTC, the primary and secondary types were determined according to the largest diameter of the nodule. Specifically, PTC with the largest diameter was classified as the primary PTC, while the other nodules were classified as secondary PTC regardless of the PTC location.

### Statistical Analysis

The data were assessed for normality using the Kolmogorov—Smirnov test. Patient age at the time of diagnosis and the nodule size were expressed as mean ± SD. The differences in mean age and mean nodule size between the 3 set of groups (isthmic PTC vs. lobar PTC, primary isthmic PTC vs. secondary isthmic PTC, and primary isthmic PTC vs. primary lobar PTC) were compared using independent sample *t*-test. Group comparisons of categorical variables were performed using the chi-square test, or using the Fisher exact test when > 20% of cells had an expected frequency <5. All statistical analyses were performed using SPSS ver. 25.0 (IBM Corp., Armonk, NY, USA) and a *p*-value of < 0.05 was considered statistically significant.

## Results

The mean age of all 249 patients with a classic type of PTC, at the time of diagnosis, was 47.7 ± 10.5 years (range, 25–86 years). The female-to-male ratio was 6.3:1.0 (86.3 vs. 13.7%, respectively). The mean tumor size was 1.1 ± 0.6 cm (range, 0.5–4.6 cm). The type of thyroid operation performed was total thyroidectomy (76.3%; 190/249) and hemithyroidectomy (23.7%; 57/249). In the 249 patients, nodules were present in the isthmic and lobar locations in 104 and 145 patients, respectively. Of the 104 patients with isthmic PTC, 95 and 9 were categorized into primary and secondary isthmic PTC, respectively, whereas all 145 patients had primary lobar PTC. Of the 9 patients with secondary isthmic PTC, the location of primary PTC was right lobe (*n* = 5), left lobe (*n* = 4), and isthmus (*n* = 0).

**Figure 2:**
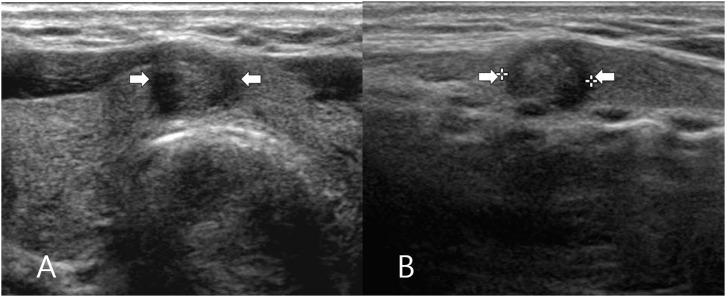
A 62-year-old woman who underwent total thyroidectomy for papillary thyroid carcinoma in the thyroid isthmus. Transverse **(A)** and longitudinal **(B)** gray-scale sonograms of the isthmus obtained preoperatively show a solid thyroid nodule with hypoechogenicity, spiculated/microlobulated margin, microcalcifications, and parallel orientation (arrows) (5.4 × 6.5 × 7.7 mm; K-TIRADS category 5). After total thyroidectomy, this nodule was diagnosed by post-operative histopathological analysis as a solitary papillary thyroid carcinoma (classic type).

**Figure 3:**
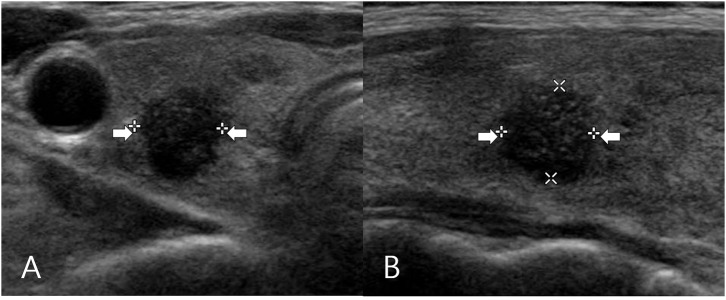
A 57-year-old woman who underwent total thyroidectomy for papillary thyroid carcinoma in the right thyroid lobe. Transverse **(A)** and longitudinal **(B)** gray-scale sonograms of the right lobe obtained preoperatively show a solid thyroid nodule with hypoechogenicity, spiculated/microlobulated margin, microcalcifications, and non-parallel orientation (arrows) (7.6 × 7.9 × 8.0 mm; K-TIRADS category 5). After total thyroidectomy, this nodule was diagnosed by post-operative histopathological analysis as a solitary papillary thyroid carcinoma (classic type).

The clinical and US findings of the classic PTC according to isthmic and lobar types are shown in Table 1. All PTC nodules had a solid composition. Isthmic PTC showed a lower prevalence of non-parallel orientation than lobar PTC (23.1 and 71%, respectively), whereas both isthmic and lobar PTC showed a high prevalence of microcalcification (69.2 and 79.3%, respectively) or spiculated/microlobulated margin (93.3 and 95.2%, respectively) (Figures 2, 3). Among the US features, only nodule orientation was statistically different between isthmic and lobar PTC groups (*p* < 0.0001). There was no significant difference in patient age, sex, nodule size, composition, echogenicity, microcalcification, spiculated/microlobulated margin, and K-TIRADS category (*p* > 0.05).

**Table 1:** Comparison of clinical and ultrasonographic findings in 249 patients with isthmic and lobar papillary thyroid carcinomas.

**Items**	**Isthmic PTC (*n* = 104)**	**Lobar PTC (*n* = 145)**	***p*-value**
Age (mean ± SD, yr)	46.9 ± 9.9	48.4 ± 10.9	0.280
Gender			0.576
Female	88 (84.6)	127 (87.6)	
Male	16 (15.4)	18 (12.4)	
Size (mean ± SD, cm)	1.1 ± 0.6	1.1 ± 0.6	0.534
Nodule echogenicity			0.696
iso-	3 (2.9)	3 (2.1)	
hypo-	101 (97.1)	142 (97.9)	
hyper-	0 (0)	0 (0)	
Specific US features			
Microcalcification	72 (69.2)	115 (79.3)	0.062
Spiculated/microlobulated	97 (93.3)	138 (95.2)	0.583
Non-parallel	24 (23.1)	103 (71)	<0.0001
K-TIRADS category			0.489
2	0 (0)	0 (0)	
3	0 (0)	1 (0.7)	
4	5 (4.8)	4 (2.8)	
5	99 (95.2)	140 (96.6)	

*Data are presented as the number of items, with the percentage in parentheses. PTC, papillary thyroid carcinoma; US, ultrasonography*.

Most cases of isthmic (95.2%; 99/104) and lobar PTC (96.6%; 140/145) belonged to K-TIRADS category 5. Five cases of isthmic PTC and 4 cases of lobar PTC belonged to K-TIRADS category 4 (Table 1): of the 5 isthmic PTC cases with K-TIRADS category 4, 2 showed solid, hypoechoic nodules with no indications of malignant US features, 1 demonstrated solid isoechoic nodule with microcalcification, and 2 showed solid isoechoic nodule with microlobulated/spiculated margin; of the 4 lobar PTC cases with K-TIRADS category 4, 2 showed solid hypoechoic nodule with no indications of malignant US features and 2 showed solid isoechoic nodule with microcalcification. The mean nodule size was 1.0 ± 0.6 cm in K-TIRADS category 5 cases (*n* = 239) and 1.5 ± 1.0 cm in K-TIRADS category 4 cases (*n* = 9). Only 1 case of lobar PTC was found belonging to K-TIRADS category 3 (nodule size, 3.4 cm).

**Figure 4:**
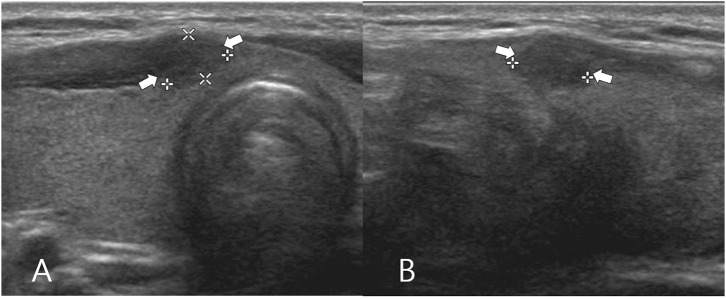
A 45-year-old woman who underwent total thyroidectomy for papillary thyroid carcinomas in the left thyroid lobe (not shown) and thyroid isthmus. Transverse **(A)** and longitudinal **(B)** gray-scale sonograms of the thyroid isthmus obtained before preoperatively show a solid thyroid nodule with hypoechogenicity, spiculated/microlobulated margin, no calcifications, and non-parallel orientation (arrows) (4.1 × 5.8 × 6.6 mm; K-TIRADS category 5). After total thyroidectomy, this nodule was diagnosed by post-operative histopathological analysis as a secondary papillary thyroid carcinoma (classic type).

The clinical and US findings of isthmic PTC according to the primary and secondary types are shown in Table 2. Both primary and secondary isthmic PTC cases showed a high prevalence of microcalcification (69.5 and 66.7%, respectively) and spiculated/microlobulated margin (93.7 and 88.9%, respectively) but a lower prevalence of non-parallel orientation (24.2 and 11.7%, respectively) (Figure 4). Comparison between primary (*n* = 95) and secondary (*n* = 9) isthmic PTC showed no significant differences in patient age, sex, nodule size, echogenicity, the three malignant US features (microcalcification, spiculated/microlobulated margin, and non-parallel orientation), and K-TIRADS category (*p* > 0.05).

**Table 2:** Comparison of clinical and ultrasonographic findings in 104 patients with isthmic papillary thyroid carcinoma categorized according to primary and secondary types.

**Items**	**Primary (*n* = 95)**	**Secondary (*n* = 9)**	***p*-value**
Age (mean ± SD, yr)	46.9 ± 10.0	47.2 ± 9.3	0.918
Gender			1.000
Female	80 (84.2)	8 (88.9)	
Male	15 (15.8)	1 (11.1)	
Size (mean ± SD, cm)	1.1 ± 0.6	1.1 ± 0.4	0.873
Nodule echogenicity			0.240
iso-	2 (2.1)	1 (11.1)	
hypo-	93 (97.9)	8 (88.9)	
hyper-	0 (0)	0 (0)	
Specific US features			
Microcalcification	66 (69.5)	6 (66.7)	1.000
Spiculated/microlobulated	89 (93.7)	8 (88.9)	0.480
Non-parallel	23 (24.2)	1 (11.7)	0.681
K-TIRADS category			0.058
2	0 (0)	0 (0)	
3	0 (0)	0 (0)	
4	3 (3.2)	2 (22.2)	
5	92 (96.8)	7 (77.8)	

*Data are presented as the number of items, with the percentage in parentheses. PTC, papillary thyroid carcinoma; US, ultrasonography*.

The clinical and US findings of classic cases of primary isthmic and lobar PTC is shown in Table 3. Among the US findings, only nodule orientation was statistically different between the two groups (*p* < 0.0001). The primary isthmic PTC group showed a lower prevalence of non-parallel orientation as compared with the lobar PTC group (24.2 vs. 71%), whereas both primary isthmic and lobar PTC groups showed a high prevalence of microcalcification (69.5 vs. 79.3%) or spiculated/microlobulated margin (93.7 vs. 95.2%). There were no significant differences in patient age, sex, nodule size, echogenicity, multifocality, microcalcification, spiculated/microlobulated margin, and K-TIRADS category between the two groups (*p* > 0.05).

**Table 3:** Comparison of clinical and ultrasonographic findings of patients with primary isthmic and primary lobar papillary thyroid carcinomas.

**Items**	**Isthmic PTC (*n* = 95)**	**Lobar PTC (*n* = 145)**	***p*-value**
Age (mean ± SD, yr)	46.9 ± 10.0	48.4 ± 10.9	0.285
Gender			0.566
Female	80 (84.2)	127 (87.6)	
Male	15 (15.8)	18 (12.4)	
Size (mean ± SD, cm)	1.1 ± 0.6	1.1 ± 0.6	0.526
Multifocality			0.493
Solitary	64 (67.4)	91 (62.8)	
Multiple	31 (32.6)	54 (37.2)	
Nodule echogenicity			1.000
iso-	2 (2.1)	3 (2.1)	
hypo-	93 (97.9)	142 (97.9)	
hyper-	0 (0)	0 (0)	
Specific US features			
Microcalcification	66 (69.5)	115 (79.3)	0.079
Spiculated/microlobulated	89 (93.7)	138 (95.2)	0.772
Non-parallel	23 (24.2)	103 (71)	<0.0001
K-TIRADS category			0.709
2	0 (0)	0 (0)	
3	0 (0)	1 (0.7)	
4	3 (3.2)	4 (2.8)	
5	92 (96.8)	140 (96.6)	

*Data are presented as the number of items, with the percentage in parentheses. PTC, papillary thyroid carcinoma; US, ultrasonography*.

## Discussion

Previous studies have reported a 1 ~ 9.2% incidence of PTC in the thyroid isthmus, which possibly reflects variations in the study populations (1–4, 7, 8). In the present study, the incidence of isthmic PTC was 6.8%, and this falls within the reported incidence range for isthmic PTC. We only included the classic type of PTC since the US features and K-TIRADS categorization of PTC may differ across PTC subtype.

To date, several TIRADS have been introduced to effectively evaluate cases of suspicious thyroid nodules (9, 12–14). Among them, the K-TIRADS is characterized by superior predictability of suspicious US features based on the solidity and echogenicity of thyroid nodules (9). Particularly, the simplicity of K-TIRADS lies in its ability to categorize thyroid nodules exclusively based on several US features (9). In the present study, we attempted to verify the diagnostic efficiency of K-TIRADS using retrospective US analysis of isthmic and lobar PTC. No significant difference was observed in the K-TIRADS categorization between isthmic and lobar PTC (*p* > 0.05). In addition, most isthmic and lobar PTC belonged to K-TIRADS category 5 (96.8 and 96.6%, respectively), whereas all the remaining isthmic PTC belonged to K-TIRADS category 4 (3.2%). Thus, we believe that K-TIRADS is useful for diagnosing both isthmic and lobar PTC.

Among the various US features examined in our study, only nodule orientation showed statistically significant difference between cases of isthmic and lobar PTC. Isthmic PTC showed lower prevalence of non-parallel orientation than lobar PTC, in congruence with previously reported results (7, 8). This observation may be associated with the specific anatomy of the thyroid isthmus. Anatomically, the thyroid isthmus is a thin structure (usually 4–6 mm), which is bounded anteriorly by the sternothyroid and sternohyoid muscles and posteriorly by the 2nd and 3rd tracheal rings (15). This restriction in space may disturb the anteroposterior growth of isthmic PTC.

Between primary and secondary isthmic PTC, there were no significant difference in the clinical and US findings, as well as the K-TIRADS category. These findings are similar to those reported in our recent study on the US features of multifocal PTC (16). Thus, it is possible that the US features and K-TIRADS categories of PTC may be similar for PTC belonging to the same subtype and location. Additional studies are required for further clarification.

This study has several limitations. First, only classic type of PTC was included. Second, small-sized PTC <0.5 cm were excluded. Third, the sample size of the secondary isthmic PTC group was small. Fourth, a limited number of US images could be examined because of the retrospective analysis. Finally, a single radiologist analyzed all US images.

In conclusion, our results demonstrate that most cases of isthmic and lobar PTC belong to K-TIRADS category 5, and only the nodule orientation parameter was different between isthmic and lobar PTC. Therefore, K-TIRADS may be useful in the diagnosis of both isthmic and lobar PTC.

## Data Availability Statement

The raw data supporting the conclusions of this article will be made available by the authors, without undue reservation, to any qualified researcher.

## Ethics Statement

The studies involving human participants were reviewed and approved by Busan Paik Hospital Institutional Review Board and Ethics Committed. Written informed consent for participation was not required for this study in accordance with the national legislation and the institutional requirements.

## Author Contributions

DWK: concept, design, and review of final manuscript. YL and DWK: acquisition of data and manuscript writing. HB and DWK: analysis and interpretation of data. All authors: literature review and refinement of manuscript.

## Conflict of Interest

The authors declare that the research was conducted in the absence of any commercial or financial relationships that could be construed as a potential conflict of interest.
